# Targeting the Immune System With Mesenchymal Stromal Cell-Derived Extracellular Vesicles: What Is the Cargo's Mechanism of Action?

**DOI:** 10.3389/fbioe.2019.00308

**Published:** 2019-11-05

**Authors:** Jorge Diego Martin-Rufino, Natalia Espinosa-Lara, Lika Osugui, Fermin Sanchez-Guijo

**Affiliations:** ^1^Unidad de Terapia Celular, Servicio de Hematología, IBSAL-Hospital Universitario de Salamanca, Salamanca, Spain; ^2^Facultad de Medicina, Universidad de Salamanca, Salamanca, Spain; ^3^Centro en Red de Medicina Regenerativa y Terapia Celular de Castilla y León, Salamanca, Spain

**Keywords:** mesenchymal stromal cells, extracellular vesicles, immune system, cell therapy, regenerative medicine, stem cells, paracrine signaling, immunomodulation

## Abstract

The potent immunomodulatory activities displayed by mesenchymal stromal cells (MSCs) have motivated their application in hundreds of clinical trials to date. In some countries, they have subsequently been approved for the treatment of immune disorders such as Crohn's disease and graft-versus-host disease. Increasing evidence suggests that their main mechanism of action *in vivo* relies on paracrine signaling and extracellular vesicles. Mesenchymal stromal cell-derived extracellular vesicles (MSC-EVs) play a prominent role in intercellular communication by allowing the horizontal transfer of microRNAs, mRNAs, proteins, lipids and other bioactive molecules between MSCs and their targets. However, despite the considerable momentum gained by MSC-EV research, the precise mechanism by which MSC-EVs interact with the immune system is still debated. Available evidence is highly context-dependent and fragmentary, with a limited number of reports trying to link their efficacy to specific active components shuttled within them. In this concise review, currently available evidence on the molecular mechanisms underlying the effects of MSC-EV cargo on the immune system is analyzed. Studies that pinpoint specific MSC-EV-borne mediators of immunomodulation are highlighted, with a focus on the signaling events triggered by MSC-EVs in target immune cells. Reports that study the effects of preconditioning or “licensing” in MSC-EV-mediated immunomodulation are also presented. The need for further studies that dissect the mechanisms of MSC-EV cargo in the adaptive immune system is emphasized. Finally, the major challenges that need to be addressed to harness the full potential of these signaling vehicles are discussed, with the ultimate goal of effectively translating MSC-EV treatments into the clinic.

## Mesenchymal Stromal cells, Extracellular Vesicles, and Immunomodulation

Mesenchymal stromal cells (MSCs) are a heterogeneous population of non-hematopoietic, fibroblast-like adult progenitor cells, which can be isolated from a variety of tissue sources (Hass et al., 2011; Samsonraj et al., 2017). MSCs can self-renew and are multipotent, given their ability to differentiate into adipocytes, chondrocytes, and osteocytes *in vitro* (Dominici et al., 2006). MSCs' ease of expansion, together with their tissue repair and immunotherapeutic capabilities, have motivated their application in hundreds of clinical trials to date and their subsequent approval in some countries as therapeutic agents for several immune disorders, such as Crohn's disease and graft-versus-host disease (Galderisi et al., 2016; Najar et al., 2016; Galipeau and Sensébé, 2018).

MSCs display potent immunosuppressive and anti-inflammatory activities, as well as low immunogenicity (Gao et al., 2016). They regulate the innate and adaptive immune systems, and target virtually all immune populations. MSCs suppress lymphocyte proliferation and activation, reduce cytokine secretion and cytotoxicity and induce peripheral tolerance and regulatory cell expansion (Di Nicola et al., 2002; Le Blanc et al., 2003; Zhao et al., 2016). They also hinder dendritic cell maturation and activation and polarize proinflammatory M1 toward anti-inflammatory M2 macrophages (Németh et al., 2009; Spaggiari et al., 2009).

Migration, engraftment and subsequent differentiation into target cells were initially considered the main mechanisms by which MSCs exert their therapeutic effects in regenerative applications. However, paracrine signaling is currently regarded as the primary mode of action of MSCs (Bi et al., 2007; Di Trapani et al., 2016; Vizoso et al., 2017; Ferreira et al., 2018). Indeed, the recent proposal to rename MSCs to “medicinal signaling cells” reflects the paradigm shift in the conception of their main mechanism of action *in vivo*, which is not multipotency but rather the production of trophic factors and bioactive molecules (Caplan, 2017). Furthermore, engraftment following infusion in recipient organisms is rare. It has been reported that MSCs are trapped in the lungs' microvasculature and are cleared within 24 h (Eggenhofer et al., 2012).

The secretome of MSCs encompasses a wide array of factors, including cytokines, chemokines, growth factors, and extracellular matrix proteins. Among the main candidates of MSCs' immunomodulatory effects are indoleamine 2,3-dioxygenase 1, prostaglandin E_2_ (PGE_2_), hepatocyte growth factor, transforming growth factor beta 1, TNF-alpha induced protein 6 (TSG-6) and major histocompatibility complex class I G (Di Nicola et al., 2002; Meisel et al., 2004; Aggarwal and Pittenger, 2005; Selmani et al., 2008; Choi et al., 2011). Extracellular vesicles (EVs)—bilipid membrane-enclosed vesicles ranging from 50 nm to a few μm in diameter—are also an important part of the MSC secretome (Lener et al., 2015). Mesenchymal stromal cell-derived extracellular vesicles (MSC-EVs) play a prominent role in intercellular communication by allowing the horizontal transfer of microRNAs (miRNAs), mRNAs, lncRNAs, proteins, lipids and other bioactive molecules between MSCs and target cells (Rani et al., 2015; Maas et al., 2017; van Niel et al., 2018).

So far, MSC-EVs have shown considerable therapeutic potential as cell-free surrogates of MSC immunomodulation in multiple disease models (Bruno et al., 2009; Lai et al., 2010). MSC-EVs have also been tested in a patient with graft-versus-host disease and in a patient cohort suffering from chronic kidney disease, although further evidence is still needed to support their widespread clinical use (Kordelas et al., 2014; Nassar et al., 2016). However, despite the considerable momentum gained by MSC-EV research, the precise mechanism by which MSC-EVs interact with the immune system is still debated, since available evidence is highly context-dependent and fragmentary, with a limited number of reports trying to link their efficacy to specific active components shuttled within them. An improved understanding of MSC-EVs' mechanism of action is therefore crucial to improve and standardize these therapies. Furthermore, by enriching MSC-EV cargo with certain bioactive molecules through specific preconditioning protocols tailored to each disease and patient, these strategies could potentially contribute to overcoming one of the major hurdles of MSC-based therapeutics, which is variable patient response (Wang et al., 2014a; Galipeau et al., 2016; Najar et al., 2018; Noronha et al., 2019).

In this work, the currently available evidence on the molecular mechanisms underlying MSC-EV cargo's immunomodulatory effects is reviewed. Although multiple reports have studied the effects of MSC-EVs on the immune system, this concise review selects and organizes studies that functionally validate and pinpoint specific MSC-EV-borne mediators of immunomodulation. The signaling events triggered by MSC-EVs in target immune cells are highlighted. Reports that study the effects of preconditioning in MSC-EV-mediated immunomodulation are also presented, as well as the major challenges that need to be addressed to harness the full potential of these signaling vehicles and effectively bring MSC-EV treatments to the clinic.

## Characterization and Cargo of Mesenchymal Stromal Cell-Derived Extracellular Vesicles

MSC-EVs have been traditionally classified as exosomes, microvesicles, and apoptotic bodies (Giebel et al., 2017). However, physical separation of MSC-EVs by this biogenesis-based classification is not realistic given the current lack of unequivocally or universally specific makers for each subtype (Lötvall et al., 2014). For this reason, a recent position statement from the International Society for Extracellular Vesicles recommends to instead refer to EV subtypes using operational terms, such as size, density, biochemical composition, or descriptions of specific EV conditions or cell of origin (Théry et al., 2018).

MSC-EV characterization should comprise multiple, complementary techniques to rule out confounding factors and assure that observed biological effects are not due to co-isolated materials (Théry et al., 2018). However, given the large number of MSC sources, culture conditions, preconditioning protocols, and EV isolation strategies, developing a one-size-fits-all solution to define MSC-EVs is not feasible. As a minimum prerequisite, MSC-EVs should derive from MSCs that meet the minimal criteria from the International Society for Cell and Gene Therapy (Dominici et al., 2006; Ramos et al., 2016; Witwer et al., 2019). MSC-EV characterization should also include assessment of MSC-EVs' lipid to protein or RNA ratios, purity, integrity, and cargo biological activity (Witwer et al., 2019).

Selective sorting mechanisms operate to enrich specific RNAs, proteins and lipids within EVs (Anand et al., 2019). Despite the aforementioned disparity of MSC-EV sources and preparation methods, a common specific MSC-EV proteomic signature has been recently proposed (Van Balkom et al., 2019). Comprehensive overviews of the miRNA and lipidomic profiles of MSC-EVs harvested from different tissue sources have also been published (Baglio et al., 2015; Fang et al., 2016; Ferguson et al., 2018; Kaur et al., 2018; Liu et al., 2019; Showalter et al., 2019). As the field of MSC-EV research grows, similar approaches will be crucial to understand and characterize the profile of EVs harvested from preconditioned MSCs.

## Molecular Mediators of the Cross-talk Between Mesenchymal Stromal Cell-derived Extracellular Vesicles and the Immune System

**Figure 1:**
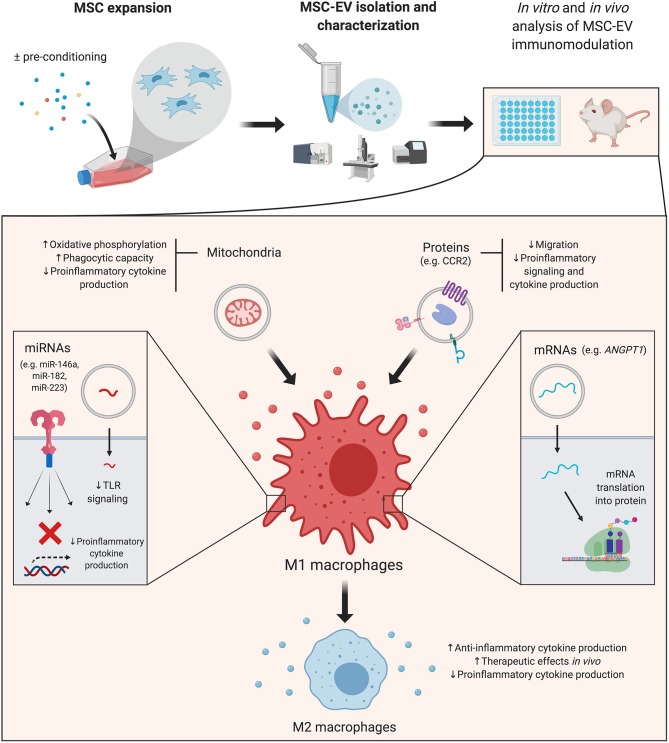
Mesenchymal stromal cell-derived extracellular vesicles (MSC-EVs): cargo's effects on macrophages. A simplified overview of the main effects triggered by MSC-EV cargo in macrophages–which are the focus of the majority of reviewed studies–is presented. MSC-EVs are isolated from cultured cells, which can be pre-conditioned with different strategies to increase their immunomodulatory potential. Details of the effector molecules, signaling pathways and mediated immunological effects are presented in the main text. *ANGPT1*, angiopoietin 1; CCR2, membrane receptor chemokine (C-C motif) receptor 2; miRNA, microRNA; MSC, mesenchymal stromal cell; MSC-EV, mesenchymal stromal cell-derived extracellular vesicle; TLR, toll like receptor. Created with BioRender.com.

The majority of reports presented here focus on macrophages (Figure 1), underscoring the need for further studies that dissect these mechanisms in adaptive immunity. Interestingly, a recent paper reported that MSCs' immunomodulatory effects on B cells were not mediated by secreted MSC-EVs (Carreras-Planella et al., 2019). It is worth noting that the authors used size-exclusion chromatography to isolate EVs, in contrast with most of the works reviewed here, which rely on ultracentrifugation methods. Previous reports have also suggested that MSC-EVs are not as effective as their cellular counterparts (Conforti et al., 2014; Gouveia-de-Andrade et al., 2015). This highlights the necessity of greater standardization and reproducibility, given the variety of cell sources, isolation and functional assays available (Théry et al., 2018).

### miRNAs

miRNAs are a subtype of small non-coding RNAs which interact with target mRNA molecules to induce their degradation and translational repression (O'Brien et al., 2018). miRNAs are selectively sorted into EVs, in which they remain protected from degradation (Li et al., 2012). Thus, miRNAs are delivered into target cells with high efficiency, in which they exert biological effects and regulate cell activity (Gallo et al., 2012; O'Brien et al., 2018). Several miRNAs have been singled out as mediators of the immunomodulatory effects of MSC-EVs on immune populations, and are briefly summarized below.

Toll like receptor 4 (TLR4) and the MYD88 innate immune signal transduction adaptor (MYD88)-dependent signaling pathway are the focus of a significant number of studies that analyze the effects of MSC-EV cargo on macrophage function. This signaling pathway mediates macrophage activation upon recognition of pathogen-associated molecular patterns such as bacterial lipopolysaccharide (LPS) (Lu et al., 2008; Park and Lee, 2013). Downstream signaling effectors converge to nuclear factor kappa B (NF-κB), which controls the expression of numerous proinflammatory molecules and drives macrophages into a M1 proinflammatory phenotype (Wang et al., 2014b). Several miRNAs presented in this and the preconditioning sections of the review target components of the TLR pathway. Among such miRNAs are miR-21, miR-146a, miR-182 or miR-223, to cite a few, which are enriched in MSC-EVs and act as negative regulators of this pathway (O'Neill et al., 2011; Qin et al., 2018; Curtale et al., 2019).

Murine MSC-EVs, enriched in miR-182-5p, have been shown to reduce TLR4, MyD88 and NF-κB levels in macrophages and induce polarization toward an M2 anti-inflammatory phenotype (Zhao et al., 2019). These effects were recapitulated by a miR-182 mimic and partially reversed upon miR-182 depletion in MSC-EVs. In an *in vivo* murine model of myocardial damage, the authors also showed that TLR4-deficient mice recapitulated MSC-EV-mediated dampening of myocardial inflammation and macrophages' phenotype shift.

An additional study focusing on macrophage polarization analyzed the role of MSC-EV-borne mir-223 (Wang et al., 2015). Through loss-of-function studies, it was shown that MSC-EVs from *Mir223* knockout mice failed to reduce LPS-induced cytokine production in macrophages. In turn, MSC-EVs from wild type mice carrying miR-223-5p and -3p inhibited this secretion. Furthermore, MSC-EVs dampened systemic inflammatory response, reduced cardiac dysfunction and increased survival in a murine model of polymicrobial sepsis, while injection of MSC-EVs from *Mir223* knockout mice failed to display these effects. Both miR-223-5p and -3p target numerous inflammatory mediators and have recently emerged as a critical factors in the pathogenesis of sepsis and inflammatory disease (Haneklaus et al., 2013).

In a report by Liu et al. (2018), mouse MSC-EVs also suppressed the secretion of proinflammatory mediators by impeding the activation of the nucleotide-binding and oligomerization domain-like receptor 3 (NLRP3) inflammasome in macrophages both *in vitro* and *in vivo*. Recently, several miRNAs have emerged as key regulators of the inflammasome, among which miR-17 is included (Zamani et al., 2019). Enriched in murine MSC-EVs, miR-17 was shown to reduce TXNIP levels, a key mediator of NLRP3 inflammasome activation. Disease amelioration and decreased NLRP3, caspase 1, IL-1ß and IL-18 levels were observed upon MSC-EV treatment. The observed *in vitro* and *in vivo* effects in macrophages were abrogated upon miR-17 knockdown in MSC-EVs.

miR-21-5p, highly enriched in human MSC-EVs, was pinpointed as a potential mediator of human MSC-EVs' impairment of dendritic cells' migration ability *in vitro* (Reis et al., 2018). MSC-EV-borne miR-21-5p decreased dendritic cells' CCR7 levels, a chemokine receptor with a crucial role in mature dendritic cell lymph node homing (Comerford et al., 2013). In this study, MSC-EVs also reduced antigen uptake in immature dendritic cells, limited their maturation and activation and increased anti-inflammatory cytokine production.

Corneal MSC-EVs were shown to reduce corneal neutrophil infiltration and fibrotic gene expression after wounding (Shojaati et al., 2019). However, MSC-EVs' regenerative capabilities were hampered following knockdown of Alix, a component of the miRNA EV-packaging system. This evidence, together with previous studies on liver fibrosis and inflammation, underscores the role of MSC-EV-borne miRNAs as mediators of MSC-EVs' interactions with the extracellular matrix, fibrosis, and scarring (Qiu et al., 2018).

### mRNAs

Horizontal mRNA transfer mediated by MSC-EVs has recently emerged as a mechanism of paracrine exchange of genetic information by MSCs (Tomasoni et al., 2013; Ragni et al., 2017). These studies demonstrated effective horizontal mRNA transfer by MSC-EVs and subsequent translation in recipient cells, thus conferring new functionality. These findings are supported by the notion that MSC-EVs display a specific mRNA profile rather than just a random subset of expressed genes (Bruno et al., 2009; Ragni et al., 2017).

In a murine model of LPS-induced acute lung injury, experimental data suggested that human MSC-EV-transferred fibroblast growth factor 7 (*FGF7*, also known as keratinocyte growth factor) mRNA could mediate the therapeutic effects of MSCs. *FGF7* mRNA transfer and subsequent translation into human FGF7 protein contributed to a decrease in neutrophil influx and chemokine (C-X-C motif) ligand 2 (CXCL2) levels in the bronchoalveolar lavage fluid (BALF) (Zhu et al., 2014). It is worth noting that *FGF7* is one the most highly enriched mRNA in human MSC-EVs (Ragni et al., 2017). Indeed, the effects on acute lung injury were partially negated following MSC pretreatment with siRNA against *FGF7*. However, in this study, whether *FGF7* mRNA directly targeted the lung epithelium or inflammatory cells remained unanswered.

In the context of MSC-EVs' immunomodulatory effects on immune populations, a subsequent study by the same group demonstrated that angiopoietin 1 (*ANGPT1*) mRNA in MSC-EVs mediated an increase in IL-10 and a decrease in TNF-alpha in macrophages *in vitro* (Tang et al., 2017). This effect was lost in EVs derived from MSCs transfected with shRNA against *ANGPT1*, thus demonstrating a role for mRNA transfer in the crosstalk between MSC-EVs and immune cells. Reminiscent of the first study, MSC-EVs also reduced neutrophil influx and CXCL2 levels in the BALF of mice with LPS-induced acute lung injury. In contrast, *ANGPT1* mRNA-deficient MSC-EVs failed to do so.

### Proteins

MSC-EVs are endorsed with functional surface proteins and may also modulate target cells by delivering intracellular proteins. Among the first group of molecules is the membrane receptor chemokine (C-C motif) receptor 2 (CCR2), enriched in murine MSC-EVs. It has been suggested that MSC-EV-borne CCR2 acts as a decoy to bind and reduce free extracellular C-C motif chemokine ligand 2 (CCL2) levels and function (Shen et al., 2016). MSC-EV treatment blocked CCL2 effects on macrophage recruitment, induction of NF-κB signaling and subsequent expression of proinflammatory cytokines.

TSG-6 was also validated as a one of the protein mediators of MSC-EV immunomodulation in a murine model of hyperoxia-induced lung injury. MSC-EV-induced decrease in neutrophil infiltration in BALF was negated by neutralizing anti-TSG-6 antibodies and by siRNA knockdown of TSG-6 within vesicles (Chaubey et al., 2018).

Inflammation-stimulated MSC-EVs were also found to be enriched in cyclooxygenase 2 (COX2), which might contribute to a local increase in PGE_2_ (Harting et al., 2018). Moreover, MSC-EVs treated with COX2 inhibitors displayed reduced anti-inflammatory capabilities in an *in vitro* TNF-alpha production assay.

### Mitochondria

Intriguingly, mitochondrial transfer to target cells has been recently identified as a mediator of MSCs' biological effects (Spees et al., 2006; Otsu et al., 2009; Islam et al., 2012; Konari et al., 2019). In particular, MSC-EV-mediated mitochondrial transfer enhanced oxidative phosphorylation in macrophages, which resulted in a reduction of their proinflammatory cytokine secretion and increased phagocytic capacity (Morrison et al., 2017). Phinney et al. (2015) provided further evidence that macrophages could engulf MSC-EV-borne mitochondria, which in turn enhanced macrophage bioenergetics. Collectively, these studies suggest a role for MSC-EV-mediated mitochondrial transfer in the modulation of the innate immune system.

## Preconditioning Modulates Mesenchymal Stromal Cell-Derived Extracellular Vesicle Cargo and Improves Its Therapeutic Effects

The immunosuppressive properties of MSCs are deeply influenced by the stimuli present in their local microenvironment, and the nature of these signals fundamentally determines the immunomodulatory effects of MSCs (Ren et al., 2008; Wang et al., 2014a; Najar et al., 2018). This context-dependency is endorsed by the fact that MSCs display increased efficacy in preclinical models of established inflammatory disease compared to their preventive administration before inflammation develops (Sudres et al., 2006; Shi et al., 2012). For this reason, several strategies have been proposed in order to precondition MSCs and improve their therapeutic potential, including proinflammatory cytokines, hypoxia, or chemical compounds (Najar et al., 2018; Noronha et al., 2019). These strategies aim to recapitulate the microenvironment to which MSCs are exposed upon infusion in patients with systemic inflammation or altered immune responses (Galipeau et al., 2016). As expected, the MSC secretome and MSC-EVs are also affected by preconditioning strategies, which modulate their cargo and immunosuppressive properties (Domenis et al., 2018; Ferreira et al., 2018; Harting et al., 2018, Showalter et al., 2019).

Multiple studies have demonstrated that preconditioned MSC-EVs display an increased ability to induce an M2 anti-inflammatory phenotype in macrophages compared to basal MSC-EVs. As such, Domenis et al. (2018) reported that priming human MSCs with proinflammatory cytokines IFN-gamma and TNF-alpha favored this effect in MSC-EVs. Specific miRNAs involved in macrophage polarization were enriched in MSC-EVs following inflammatory preconditioning, among which stood miR-34a-5p, miR-146a-5p, and miR-21-5p. Interleukin 1 receptor associated kinase 1 (IRAK1), a signaling mediator of the MyD88 signaling pathway targeted by miR-146a, was strongly downregulated upon treatment with preconditioned MSC-EVs. Further functional studies will help understand the individual contribution of each identified miRNA to the observed effects.

Human MSC-EV-borne miR-146a was also the focus of Song et al. (2017), who observed that this miRNA was particularly enriched in IL-1ß-primed MSC-EVs. Primed MSC-EVs delivered miR-146a into macrophages, increased M2 markers IL-10 and *Arg1* mRNA, and decreased M1 markers TNF-alpha and *Nos2* (iNOS) mRNA in a greater extent that basal MSC-EVs. These effects were lost in IL-1ß-primed MSC-EVs previously transfected with miR-146a inhibitors, underscoring the role of miR-146a in M2 polarization by IL-1ß-primed MSC-EVs.

Increased M2 polarization capacity was also observed in EVs from LPS preconditioned MSCs. In this regard, the work from Ti et al. (2015) uncovered let-7b as the highest uniquely-expressed miRNA in human MSC-EVs with this priming strategy. let-7b regulated macrophage polarization through TLR4 and NF-κB subunit p65 downregulation and signal transducer and activator of transcription 3 (STAT3) and AKT upregulation.

Various studies focused on the influence of hypoxia in the mechanism of action of MSC-EV cargo. Lo Sicco et al. (2017) demonstrated that EVs from human MSCs cultured under hypoxic conditions displayed an increased ability to induce murine M2 anti-inflammatory macrophages compared to basal MSC-EVs. miR-223 and miR-146b stood out among over-expressed miRNAs in hypoxia-preconditioned MSC-EVs. Ren et al. (2019) provided further evidence that hypoxia-preconditioned human MSC-EVs favor human macrophage M2 polarization in comparison to their normoxic counterparts. In their study, they showed that miR-21-5p knockdown abrogated these effects, likely mediated by phosphatase and tensin homolog (PTEN) downregulation, which in turn released its blockage of AKT and STAT3 activation. These effects were maintained *in vivo* and partially mediated by miR-21-5p, which had been previously shown to be enriched in hypoxia-preconditioned murine MSC-EVs (Cui et al., 2018). Hypoxia is one of the hallmarks of tissue damage, and its use in MSC preconditioning might mimic the microenvironment to which MSCs are exposed *in vivo* (Ferreira et al., 2018).

## Closing Remarks: The Road Toward Clinical-Grade, Cell-Free Therapeutics

The generalized use of clinical-grade MSC-EVs is still far from becoming a reality, based on a number of challenges that need to be solved in the coming years. One of the main limitations for the use of EVs is the low yield of particles obtained with current technologies used for EV isolation and purification, resulting in insufficient EV numbers for clinical purposes (Haraszti et al., 2018; Paganini et al., 2019). The use of spinner-flasks containing different microcarriers that retain MSCs is one of the most extended options to increase MSC numbers, together with the use of hollow-fiber bioreactors, which can anchor up to 100-fold higher MSCs than conventional flasks (Lu et al., 2017; Cha et al., 2018; Phan et al., 2018). Alternative EV isolation techniques, some of which can be scalable and potentially GMP compatible, can potentially increase MSC-EV recovery (Watson et al., 2018, Paganini et al., 2019).

The multiple immunomodulatory signaling capabilities presented in the previous sections show MSC-EVs' immense potential for the treatment of immune disorders. MSC are considered advanced-therapy medical products (ATMP) by regulatory agencies (Lener et al., 2015). In contrast, given their cell-free nature, MSC-EVs are not subject to complex ATMP regulations and do not carry the risk of carcinogenic potential transformation. Due to their size, they are able to traverse the blood-brain barrier and other physiological interfaces. Finally, MSC-EVs are easily handled and stored and remain stable for extended periods of time (Rani et al., 2015; Maas et al., 2017; Sharma et al., 2017). Such potential advantages make MSC-EVs promising candidates for future therapeutic applications.

The studies summarized in this work support the claim that the fundamental mechanism of action of MSC-EVs lies in their ability to transmit biological information between cells. Although multiple candidates within MSC-EV cargo have been presented as mediators of their therapeutic effects, many operate through similar pathways and mechanisms of action, warranting further investigations that dissect their individual contribution. These studies are the starting point for the future development of engineered MSC-EVs and targeted preconditioning protocols, which could potentially enrich MSC-EVs with relevant immunomodulatory effectors and thus broaden their therapeutic capabilities in immune system disorders.

## Author Contributions

JM-R and FS-G conceptualized, designed and wrote the manuscript. JM-R drafted the manuscript, reviewed the literature and designed the figure. NE-L and LO revised and provided critical feedback on the manuscript.

### Conflict of Interest

The authors declare that the research was conducted in the absence of any commercial or financial relationships that could be construed as a potential conflict of interest.
